# Antidepressant Effects of a Persian Medicine Remedy on Multiple Sclerosis Patients: A Double-Blinded Randomized Clinical Trial

**DOI:** 10.31661/gmj.v8i0.1212

**Published:** 2019-01-01

**Authors:** Maryam Adalat, Mohammad Khalili, Hormoz Ayromlou, Sajjad Haririan, Seyyed Mohammad Bagher Fazljou, Hossein Rezaeizadeh, Ali Akbar Safari, Arman Zargaran

**Affiliations:** ^1^Department of Traditional Medicine, School of Traditional Medicine, Tabriz University of Medical Sciences, Tabriz, Iran; ^2^Multiple Sclerosis Research Center, Neuroscience Institute, Tehran University of Medical Sciences, Tehran, Iran; ^3^Neurosciences Research Center; Tabriz University of Medical Sciences, Tabriz, Iran; ^4^Department of Neurology, Tabriz University of Medical Sciences, Tabriz, Iran; ^5^Department of Neurology, Alinasab Hospital, Tabriz, Iran; ^6^Department of Traditional Medicine, School of Persian Medicine, Tehran University of Medical Sciences, Tehran, Iran; ^7^Department of Traditional Pharmacy, School of Persian Medicine, Tehran University of Medical Sciences, Tehran, Iran

**Keywords:** Persian Medicine, Multiple Sclerosis, Depression, Herbal Extract, Alternative Medicine

## Abstract

**Background:**

Multiple sclerosis (MS), an inflammatory neurodegenerative disease of the central nervous system, is accompanied by some psychiatric disorders, one prominent example of which is depression. The aim of this study was to investigate the effects of a Persian herbal medicine treatment that contains *Crocus sativus*, *Hypericum perforatum*, *Cinnamon verum*, and *Vitis vinifera* on fatigue and sleep disorders in MS patients.

**Materials and Methods:**

A Persian medicine remedy containing *C.sativus*, *H.perforatum*, *C.verum*, and *V.vinifera* was tested for its ability to improve the symptoms of depression in MS patients. This randomized double-blind clinical study was performed among 52 patients with MS who were allocated to their respective research groups through blocked randomization. The patients were treated for 4 weeks with either the drug or the placebo. To quantify the symptoms of depression, Beck depression inventory (BDI) was used.

**Results:**

Forty-six patients completed the study. In the course of the study, as the primary outcome, BDI decreased in the drug group (*p* =0.000) and the placebo group (*p* =0.001) significantly, but the rate of change in the drug group was significantly higher than in the placebo group (-13.9 ± 8.6 vs. -3.9 ± 4.3, *p* =0.000). While analyzing time and treatment effect for BDI, significant decreases in BDI were observed for the drug group, but not in the placebo group (*p* = 0.001).

**Conclusion:**

The present study suggests that Persian medicine remedy treatment in combination with chemical drugs may improve depression symptoms in MS patients. More investigations are needed to discover the exact mechanisms and processes involved.

## Introduction

Multiple sclerosis (MS) is an inflammatory and neurodegenerative disease of the central nervous system (CNS) which causes disability [1, 2]. MS is one of the most common neurologic disorders that is associated with comorbid depression, which, in turn, increases the risk of suicide [3, 4]. Studies have shown that MS patients have higher rates of depression than is the case with other chronic neurological conditions [5]. There have also been reports that genetic associations, reactive stress following diagnosis, and structural changes in the brain and neuro-inflammatory disease processes are important factors playing roles in the high prevalence of depression in MS patients [1, 6]. Despite the fact that treating depression in MS patients improves adherence to therapy with interferon β-1b, depression is generally under-diagnosed in MS patients [3, 6]. The current treatments for depression have proven to be effective, but compliance with these conventional antidepressants is often low with usually some side effects, which is one of the reasons for the use of complementary and alternative medicine (CAM) in MS patients [7, 8]. The most common CAM method used by MS patients is herbal supplementation [8]. Herbal drugs, in comparison to conventional pharmacotherapies, are fairly safe and tolerable, with fewer side effects and higher compliance rates [9, 10]. Persian medicine is one of the traditional medicine rooted in antiquity (at least 7000 years ago), practiced nowadays in Iran [11]. One of the formularies for depression, according to the *Makhzan al-Advieh* (storehouse of medicaments), a Persian medicine text on herbal medicine written by Aghili Khorasani (18th century) contained *Crocus sativus* L., *Hypericum perforatum*L., *Cinnamomum verum* J.Presl, and *Vitis vinifera* L [12]. St John’s-wort (*H.perforatum*; from the Hypericaceae family) has been known for its antidepressant activity, which is attributed to several bioactive components in it like hyperforin, hypericin and pseudohypericin [13]. It is the most popular herbal antidepressant agent used in traditional medicine for the treatment of depression [14]. The brown bark of cinnamon tree (*C.verum*; from the Lauraceae family) has commonly been used as a spice and a traditional medicine [15, 16]. The results from many clinical studies have shown that cinnamon possesses antioxidant, anti-inflammatory, anti-microbial and wound-healing abilities, thanks to its various bioactive compounds like cinnamaldehyde [15, 16]. The neuroprotective effects of the grape (*V.vinifera*; from the Vitaceae family) on neurodegenerative diseases are now well recognized; they are often attributed to high levels of bioactive compounds in grapes, including polyphenols like proanthocyanidins and resveratrol [17]. Resveratrol, one of the major polyphenolic compounds found in grapes, is present in the skin and seeds of grapes, carrying anti-inflammatory and antioxidant effects [18]. Saffron (*C.sativus*; family of Iridaceae), the dried stigma of crocus sativus blossom, is effective for the treatment of mild to moderate depression. The antidepressant effects of saffron are comparable to imipramine and fluoxetine [19, 20]. Building on the suggestions made in Persian medicine in reference to this remedy as well as the support offered by current findings as to the probable effects of its ingredients, the present study was performed to evaluate the antidepressant effects of this Persian medicine remedy containing *C.sativus*, *H.perforatum*, *C.verum*, and *V.vinifera* on patients with MS.

## Materials and Methods

### Study Design and Ethical Issues

This randomized, double-blind, placebo-controlled trial study was performed in Sina Hospital in the capital of Eastern Azarbaijan, Tabriz, Iran. The study was approved by the Ethics Committee of Tabriz Medical University (code: TBZMED.REC.1394.884), with all participants providing an informed written consent. This clinical study was registered in Iranian Clinical Trial Registry (code: IRCT2016012916369N3).

### Drug and Placebo Preparation

The plants of St John’s-wort (Voucher No. PMP-389) and cinnamon (Voucher No. PMP-913) and also the grape syrup was purchased from a traditional herbal store (*Attari*) in Tehran; their identification and quality control were carried out in the Herbarium Center of School of Pharmacy, Tehran University Medical Sciences. Standard saffron (Saharkhiz Co., Iran) was used in the study. For preparing each 10 ml of this Persian medicine remedy, the extracts of 64 mg saffron (via maceration), 357 mg cinnamon (via Soxhlet) and 857 mg St John’s-wort (via Clevenger) were obtained and put in the 4.3 ml grape syrup. Then the volume of the mixture was increased to 10 ml by adding about 2g sugar and distilled water. The placebo was prepared as a simple syrup, 0.1% St John’s-wort essential oil and 0.71 ml grape syrup to reach a color and smell similar to the drug. Both the drug and the placebo were prepared at the department of traditional pharmacy, school of Persian medicine, Tehran University of Medical Sciences.

### Inclusion and Exclusion Criteria

Eligible patients were 18 to 50 years old, with clinically definite MS disease according to McDonalds *et al*.’s criteria [21]. Inclusion criteria were considered to be: an Expanded Disability Status Score (EDSS) of less than or equal to 6, no disease attack over the previous month, no history of other autoimmune diseases and the presence of depression symptoms quantified by means of Beck Depression Inventory (BDI). Participants had to have regular contact with a responsible caregiver. Exclusion criteria were: 1) other serious psychological disorders including dementia and psychosis; 2) cardiovascular problems; 3) diabetes; 4) severe depression; 5) clinically significant major infections; 6) pregnancy and breastfeeding status; and 7) current smokers. All patients were under treatment by Selective Serotonin Reuptake Inhibitors (SSRIs). Antidepressant drugs and disease-modifying medications were maintained at a stable dose for 1 month prior to and throughout the trial. Any other herbal medications or natural antioxidants being consumed to treat depression had to be discontinued.

### Intervention

**Figure 1:**
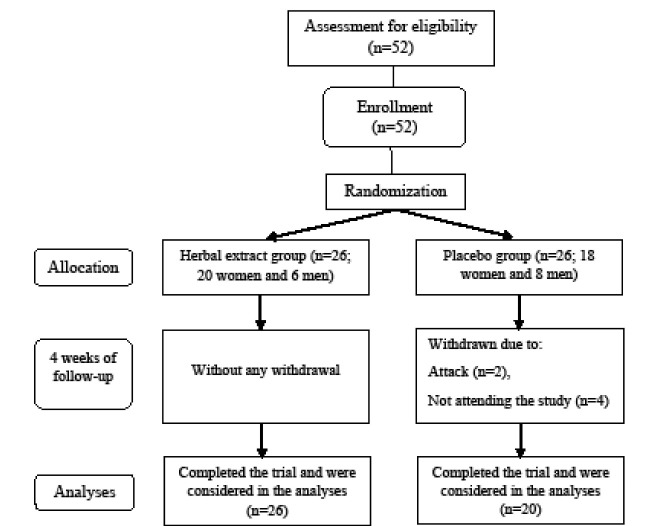
Flow diagram of the patients

Regarding inclusion criteria, fifty-two MS patients were randomly allocated by computer-generated random numbers to either an active treatment (drug) or a placebo group (ratio 1:1). Among the participants, six patients (4 men and 2 women in the placebo group) were excluded due to relapse during the study or dissatisfaction. The study was completed by 26 patients in the drug group (the treatment group) and 20 patients in the placebo group (Figure-1). Drug and placebo preparation were conducted by the pharmacological group in the Department of Persian Medicine of Tehran University of Medical Sciences, Iran. The patients in the intervention group were treated with 10 milliliters of the herbal extract twice a day for 4 weeks, whereas the control group received the placebo. All study personnel and patients were unaware of study assignments. Adherence to study medications was monitored by means of periodic telephone follow-ups between assessments.

### Outcomes

The Beck depression inventory (BDI) [22] was used to assess the level of depression in the present study. BDI consists of 21 items with scores on a four-point scale from zero (absent) to three (severe). Patients choose one statement from each item that describes how they have felt over the past week up to now. The score range is 0 to 63 and the total score is the mean of the rating from the 21 items. The BDI score was evaluated at the baseline and 4 weeks later as primary outcome.

### Statistical Analyses

Values were expressed as mean ± standard deviation (SD). Comparisons between groups (herbal extract and placebo) were performed using independent *t* test. Changes within groups were determined by Paired-sample *t* tests. Statistical comparisons were made between mean scores for the placebo group and mean scores for the herbal extract group using the repeated-measures analysis of variance (ANOVA). This ANOVA model included terms of time effect and interaction of time and treatment within the main effect (herbal extract vs. placebo). We used SPSS version 16 (SPSS Inc., Chicago, IL, USA) for statistical analyses and P < 0.05 was considered to be statistically significant.

## Results

Forty-six patients with MS completed the study. Their tolerance of the drug and placebo was desirable. No serious adverse effects were reported. At baseline, there were no significant differences between the groups in mean disease duration and mean age. The mean ± SD of baseline age was 36.8 ± 7.4 years in the drug group and 35 ± 9 years in the placebo group (*p* ˃ 0.05). No significant differences were found between the two groups regarding their marital status and gender according to demographic information (Table-1). Depression severity was evaluated at baseline and endpoint of the study. Baseline depression scores were not significantly different between the two groups. Changes of BDI within the drug group and the placebo group were observed to be a significant decrease (*p*=0.000 and *p*=0.001, respectively), but the change rate in the drug group was significantly more than the placebo group (-13.9 ± 8.6 vs. -3.9 ± 4.3, *p* =0.000, Table-2). In addition, a comparison of changes between the groups with regard to time and treatment effect using repeated measure ANOVA showed that those who were treated with the herbal extract had significantly reduced BDI scores compared to those treated with the placebo (p= 0.001). This effect was significant even after adjusting for confounding factors including age, gender and disease duration (p= 0.001, Table-2). According to the distribution of depression score changes, the 50th percentile of changes was considered as cut-off for clinically significant changes within groups, in which BDI score change-rates higher than 7 in the drug group and the placebo group were 65.4 and 10 percent, respectively, and the difference between the two groups was significant (*p*= 0.003, Table-3).

## Discussion

Depression, the most prevalent psychological consequences of multiple sclerosis, leads to both declines in the quality of life and adherence to treatment in patients [3]. Our study provided insights into the antidepressant effects of a Persian herbal extract containing *C.sativus*, *H.perforatum*, *C.verum*, and *V.vinifera* in patients with MS. The current study was the first of its kind to evaluate antidepressant effects of a combination of several herbal extracts in MS patients. Previous studies have used each of these herbal extracts individually in other depressive conditions. Our results have shown that the herbal extract significantly improved mood after 4 weeks of treatment according to BDI measures in the intervention group. The reduction in BDI measures in the intervention group was significant, even after adjustments for age, disease duration and marital status. In our trial, we studied a combination of some herbal plants in a mixture which was used to treat depression in Persian traditional medicine. When herbal plants are used in combination, their bioactive components work in synergy. This synergistic character has been used in traditional medicine for centuries and, nowadays, the use of a combination of herbal plants has become accepted practice [9, 23]. The antidepressant effects of each of the herbal plants used in this trial have been studied separately in other studies. *H.perforatum* has become a popular antidepressant in the treatment of mild to severe forms of major depression [24]. Some studies have investigated the effects of *H.perforatum* on human depression in comparison to placebos and other synthetic drugs. Helmut Woelk showed that the administration of *H.perforatum* extract (250 mg twice daily) for 6 weeks in patients with mild to moderate depression, compared to imipramine (75 mg twice daily), resulted in equivalent therapeutic effects [25]. The evaluation of the effects of *H.perforatum* compared to paroxetine in patients with acute major depression demonstrated that *H.perforatum* extract is as effective as paroxetine and is better tolerated [26]. Lecrubier *et al*. observed that the treatment of mild to moderate depressive patients with *H.perforatum* extract was safe and more effective than the placebo [27]. The underlying mechanisms of action of *H.perforatum* on depression have been proposed in several studies. *H.perforatum* and its bioactive component hyperforin inhibits the reuptake of serotonin, epinephrine and dopamine [28]. Other reports demonstrate a down-regulatory activity of *H.perforatum* on β-adrenergic receptors and up-regulatory activity on serotonin receptors in rats’ frontal cortex [29]. The release of glutamate from nerve terminals purified from rat cortex was seen to be inhibited by hypericin [30]. *H.perforatum* can affect sigma-1 receptors in a manner similar to other antidepressant drugs in animal studies. In addition, the antidepressant effect of *H.perforatum* may be due to its synergistic therapeutic effects, performed by all bioactive constituents in the whole plant working on the central nervous system [10].

A review article reported lowered concentrations of antioxidant enzymes and their activities in depression and animal models of depression, accompanied by increased levels of oxidative stress metabolites [31]. *C.verum* extract, another constituent of our herbal extract, has strong antioxidant and anti-inflammatory properties [15]. Roussel and colleagues showed that supplementation with an aqueous extract of *C.verum* (250 mg twice per day) for 12 weeks in people with impaired fasting glucose significantly reduced plasma malondialdehyde (MDA) concentration as a marker of oxidative stress [32]. Oral administration of 200 mg/kg of cinnamon extract for 7 days in rats with induced hepatic injury reduced MDA levels and increased antioxidant enzyme activities of their livers [33]. Since oxidative stress is an important factor in the pathogenesis of depression, cinnamon might have an antidepressant activity through its ability to reduce oxidative stress in patients. Another possible mechanism may involve up-regulation of neurotrophic factors by cinnamon and sodium benzoate (a metabolite of cinnamon), via activation of protein kinase A (PKA) [34]. It has been demonstrated that depression is characterized by decreased levels of brain-derived neurotrophic factor (BDNF) [35]. *V.vinifera* extract, present in our herbal extract, contains polyphenols known to have antioxidant and anti-inflammatory effects. Some of these polyphenols including resveratrol and proanthocyanidin also have neuroprotective and antidepressant properties [36-39]. Some animal studies have examined the effects of these polyphenols on depression. Hurley *et al*. showed that supplementation with resveratrol (10 and 40 mg/kg, intraperitoneal) after single dose or following 7 days of daily treatment in Wistar-Kyoto rats (animal model of depression) significantly decreased forced swim test (FST: a measure of helplessness) and increased BDNF levels in a dose-dependent manner [36]. The supplementation of rats after middle cerebral artery occlusion (model of post-stroke depression) with resveratrol led to the amelioration of decreased BDNF levels after treatment [37]. Another mechanism by which resveratrol could provide antidepressant action involves the enhancement of levels of serotonin and noradrenaline and inhibition of monoamine oxidase activity [40]. One study showed positive effects of resveratrol on FST and MDA levels in a rat model of depression. They suggested that resveratrol exerts its antidepressant effect through its peripheral effect on the regulation of hypothalamic-pituitary-adrenal (HPA) axis [41]. In addition, both proanthocyanidin and resveratrol could cross the blood-brain barrier [39, 42]. Thus, the antidepressant effects of our herbal extract could be attributed, to some extent, to the polyphenolic content of *V.vinifera*. Another constituent of the herbal extract used in this study was saffron (*C.sativus*). It has been shown that saffron has anti-depressive activity similar to synthetic antidepressant drugs such as fluoxetine and imipramine, without the attendant side effects [19, 20]. A meta-analysis review of clinical trials by Hausenblas concluded that supplementation with saffron can ameliorate depression symptoms in adults with major depressive disorders [43]. This antidepressant activity of saffron can be attributed to safranal and crocin which could inhibit dopamine, norepinephrine and serotonin uptake [44]. Saffron has antioxidant, radical scavenging and anti-inflammatory effects whereby it could inhibit oxidative stress and leukocyte infiltration to CNS in animal models of MS disease and may be useful for the treatment of multiple sclerosis [45]. Moreover, Nam *et al*. demonstrated that crocin and crocetin, found in stigmas of saffron, produce neuroprotection by reducing tumor necrosis factor-α, interleukin-1β, intracellular reactive oxygen species and nitric oxide release from activated cultured microglial cells in rat brain [46]. As previous studies have already discussed the anti-depressive properties of each herbal plant constituting our herbal extract, we conducted this trial to evaluate the synergistic effects of the combination of these herbal plants in depressed MS patients. Taken together, this study suggests that the herbal extract treatment is an efficient anti-depressive agent in MS patients. These anti-depressive effects observed in this study and the synergistic effects of herbal extract in improving depression in MS patients could be due to several bioactive compounds present in the mixture, but its exact mechanisms of action have not been established yet. The exact mechanisms involved in anti-depressive effects of herbal extracts should be further investigated.

One of the major limitations of this study is that it is not known which component is responsible for the anti-depressive effects observed in MS patients, as the herbal extract consists of complex mixtures of major and minor bioactive compounds involved in the synergistic effects. What is recommended for future research is to identify the major bioactive components of each herb and investigate the effects of each herb alone with different therapeutic doses. Another limitation is the relatively small number of patients and the short period of intervention. A trial with a longer period of intervention should be undertaken in the future.

## Conclusion

Combinations of herbal extracts with conventional drugs would be helpful in overcoming depression symptoms in patients. It has been shown that combination therapy is more effective in the treatment of depressed patients than isolated components in the mixture. The results of our study appear to suggest that this Persian medicine remedy improved depression symptoms in MS patients, probably via its bioactive components. What should be stated in conclusion is that a combination of *C.sativus*, *H.perforatum*, *C.verum*, and *V.vinifera* in addition to chemical drugs could be more useful therapeutically in improving depression symptoms in MS patients by virtue of their multiple therapeutic activities.

## Conflict of Interest

There is no conflict of interest.

**Table 1:** Basic Characteristics of Study Subjects. Values Are Presented as Mean ± Standard Deviation.

**Variable**	**Herbal group** **(n=26)**	**Placebo group** **(n=20)**	**P-value**
**Mean age (years)**	36.8 ± 7.4	35 ± 9	0.811
**Mean disease duration (years)**	7.1 ± 5.8	12 ± 5.6	0.072
**Gender**			0.511
Male	6 (23.1 %)*	4 (20%)*	
Female	20 (76.9 %)*	16 (80%)*
**Marital status**			0.112
Married	22 (84.6 %)*	4 (20%)*	
Single	4 (15.4 %)*	16 (80%)*

* Values are presented as number (%)

**Table 2:** BDI Scores at Baseline and Endpoint of Study. Values Are Presented as Means ± SD

	**Herbal group (n=26)**				**Placebo group (n=20)**			
	**Baseline**	**Endpoint**	**Change**	**P-Value**	**Baseline**	**Endpoint**	**Change**	**P-value**
**BDI score**	35 ± 9.6	21.08 ± 8.2	- 13.9 ± 8.6	<0.001*	34.5 ± 9.6	30.6 ± 10.6	-3.9 ± 4.3	0.001*

*Indicates within-group differences by paired-sample t-test

**Table 3:** Number of BDI Score Changes According to 50th Percentile of Scores after Intervention.

	**Herbal group (n=26) **	**Placebo group (n=20)**	P-Value*
**BDI score change ≥ 7**	9 (34.5%)	18 (90%)	0.003
**BDI score change < 7**	17 (65.5%)	2 (10%)	

Values are presented as number (%) *Indicates within-group differences by Chi-Square test
